# Soil organic carbon dynamics: Influences of land-use change in natural and plantation forests of the Western Ghats, India

**DOI:** 10.1371/journal.pone.0342399

**Published:** 2026-02-10

**Authors:** Panchami Jaya, Yichao Rui, M. Navya, S. Sandeep

**Affiliations:** 1 Soil science Department, KSCSTE - Kerala Forest Research Institute, Thrissur, Kerala, India; 2 Department of Agronomy, Purdue University, West Lafayette, USA,; 3 Forest Research Institute (FRI), Dehradun, Uttarakhand, India; 4 Cochin University of Science and Technology, Cochin, India; Forest Survey of India, INDIA

## Abstract

Soil organic carbon (SOC) is a fundamental component of the global carbon cycle, underpinning ecosystem health, climate regulation, and sustainable land management worldwide. The conversion of natural forests to plantation systems in humid tropical regions has emerged as a critical global issue, leading to significant reductions in SOC stocks and compromising the carbon sequestration potential of soils. To assess these impacts, we compared SOC concentrations in plantation and natural forests across five humid tropical zones, including the globally significant Southern Western Ghats (SWG) of India- a recognized biodiversity hotspot and one of the world’s most complex forest ecosystems. A stratified random sampling design was used across five agroecological zones to select paired natural forests and adjacent long-rotation teak (*Tectona grandis*) plantations (40–50 years old). Soil samples were collected from four horizons (O, A, B, and C) within 1 m depth profiles. SOC concentration (CHNS analyzer), bulk density, texture (hydrometer method), cation exchange and pH were determined. SOC stocks were calculated using bulk density and horizon depth. Our analysis shows that natural forests maintain substantially higher average SOC concentrations (16.61 g/kg) than plantation forests (11.82 g/kg). In natural forests, SOC ranged from 9.53 g/kg to 26.09 g/kg, while plantation forests ranged from 6.93 g/kg to 17.73 g/kg, reflecting similar trends observed in the SWG and other tropical regions. SOC concentrations were significantly greater in the surface layers of natural forests compared to deeper layers (P < 0.05), with more than 70% of SOC typically stored in the upper 30 cm. Correlation analysis showed a significant negative relationship between SOC and soil pH in natural forests (r = −0.37, P < 0.05), whereas plantation soils exhibited a positive relationship (r = 0.03, P < 0.05). Forest soils also showed a positive correlation between SOC and clay content (r = 0.16, P < 0.05) and a weak negative correlation with sand content (r = −0.04, P < 0.05). These findings underscore a global challenge: land use change from natural forest to plantation reduces SOC stocks, alters soil health, and diminishes the resilience of tropical soils to environmental change. Maintaining and restoring natural forests—both globally and in biodiversity hotspots like the SWG—is essential for maximizing soil carbon sequestration, supporting soil fertility, and achieving climate mitigation targets. This study provides a scientific foundation for sustainable land management and carbon storage strategies in tropical regions globally.

## 1. Introduction

Earth’s climate has never been static; it has continuously changed over millennia and centuries. Some plant and animal species go extinct, while others emerge or adapt. What is truly new, however, is the extent of human-driven interventions that are accelerating these changes. These concerns have led to widespread and intensive research efforts aimed at understanding how to stabilize the climate. Soil organic matter dynamics, associated carbon sequestration, and how different ecosystems respond to these processes form one of the key pillars for addressing the growing global concern [1–3].

Soil stores 3.3 times more carbon than the atmosphere and vegetation combined. Current studies indicate that it can store around 2500 Pg of carbon, making soil one of the major terrestrial carbon pools and a key focus in climate change studies [4]. Global soils are expected to lose as much as 50 billion metric tons of carbon by 2050. This decline in soil organic carbon (SOC) is driven largely by climate change, with additional strong impacts from land-use change and management practices that modify SOC level [5,6]. Soil organic matter (SOM) is the backbone of soil and consists of naturally complex material, including plant and animal tissues at various decomposition stages, soil microbes, and the substances they produce [7]. Despite its complexity, it is a key indicator of soil health, as it influences nutrient cycling, long-term soil fertility moisture retention and overall ecosystem functioning [7,8]. SOM classify into several pools and fractions with varying decomposition rates and stability, which help study short-term and long-term effects of land use and management on SOC dynamics [9]. Soil organic carbon (SOC) as a sensitive indicator to study how land use changes and management practices affect soil quality and SOM over time because high SOC levels correlate with improved nutrient supply to crops, soil physical properties, and biological activity. SOC balances carbon additions and losses over the long term [10,11].

In terrestrial ecosystems, forests represent the most significant global carbon pool [12]. Forest soils store more carbon than the trees themselves; however, global carbon studies have mainly focused on wood biomass and offsetting effects. Recognizing the importance of soil carbon, recent studies increasingly focus on forest soils [12,13]. Nevertheless, studies remain sparse in the Southern Western Ghats, a biodiversity hotspot that houses some of the world’s most pristine tropical forests and managed forest plantations [14]. Teak (*Tectona grandis* Linn. f) dominates forest plantations in Kerala and plays a vital role in the tropical hardwood sector. Kerala established teak plantations on a large scale since 1844, with major expansions between 1960 and 1988 under five-year plans. Besides large forest department plantations (~75,000 ha), farmers widely plant teak, managing it alongside other farm land uses. Once known as the world’s first teak plantation site and the “Mecca of teak,” Kerala faces productivity declines due to continuous monoculture with the same species, which reduces soil buffering capacity and causes chemical degradation [15].

Until now, no systematic efforts have compared long-term soil carbon dynamics between managed forest tree plantations and natural forest systems in the Southern Western Ghats. This research aims to fill critical knowledge gaps concerning SOC dynamics under different forest management systems in tropical montane environments. It has two objectives: (1) to assess and compare SOC levels in soils from natural and plantation forests in Kerala’s Southern Western Ghats; and (2) to investigate associations between SOC concentrations and key soil properties, specifically pH and texture.

## 2. Materials and methods

### 2.1. Study area

The South Western Ghats Montane Rain Forests ecoregion is the most species-rich in South Asia and is high in endemism. The northeast and southwest monsoons govern the region’s climate. The northeast monsoon provides approximately 25% of the annual rainfall, while the south west monsoon and summer showers provide the remaining 75%. The Southern Western Ghats support a wide variety of vegetation types due to their unique geographic location, stable geology, and specific soil characteristics, combined with biogeographic and climatic influences [16]. These vegetation types include tropical evergreen and semi-evergreen forests, montane rainforests, tropical moist forests, dry deciduous forests, and grasslands

**Fig 1:**
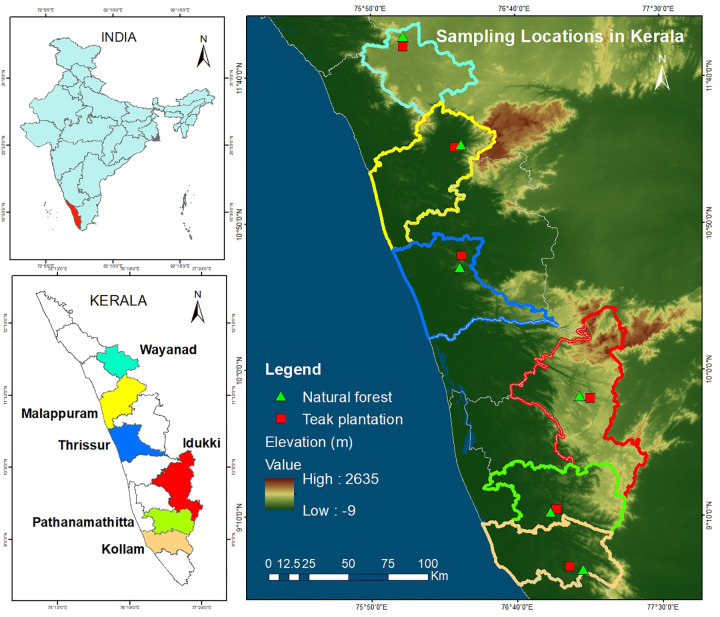
Maps of natural forest and teak planted areas of Southern Western Ghats. DEM based on Shuttle Radar Topography Mission (SRTM) 1 Arc-Second Global courtesy of the **U.**S. Geological Survey (https://doi.org/10.5066/F7PR7TFT) (https://earthexplorer.usgs.gov).

Our study area is located within the Southern Western Ghats, covering latitudes 8°–13° N and longitudes 74°–77° E, The teak plantations and natural forest were found distributed in regions with four major parent materials: charnockites, khondalites, granite – gneiss and hornblende – biotite – gneiss. Under this six representative agroecological units were selected. North Central Laterites (NCL), Southern and Central Foothills (SCF), Wayanad Central Plateau (WCP), Southern High Hills (SHH), Northern High Hills (NHH) based on soil type, altitude and climate as illustrated in Fig 1. The parameters taken for agro-ecological classification included soil characteristics, bio-climatic types, physiographical features, and length of the growing period. The Geological Survey of India (GSI), the French Institute of Pondicherry, and the ICAR-National Bureau of Soil Survey and Land Use Planning (ICAR-NBSS & LUP) prepared maps to identify geological features, forest types, and agro-ecological units, respectively (Table 1).

**Table 1:** Physiographic and geographical characteristics of the sampling sites.

Agro ecological unit	Land use type	Parent material	Location	Elevation (m)	Average rainfall (mm/year)	Temperature (°C)	Soil Order *
SCF	Teak	Khondalites	77°2’32.676“E 8° 51’ 40.557“N	270	1800	27.5	Inceptisol
SCF	Forest	Khondalites	77° 2’ 32.46“E 8° 51’ 44.46“N	295	1800	27.5	
SCF	Teak	Khondalites	76° 52’ 54.192’‘ E 9° 14’ 48.444’‘ N	265	1800	26.5	Inceptisol
SCF	Forest	Khondalites	76° 52’ 53.904’‘ E 9° 14’ 4.884’‘ N	280	1800	26.5	
SHH	Teak	Granite gneiss	77° 3’ 37.155“E 9° 44’ 58.2“N	774	2500	21.6	Inceptisol
SHH	Forest	Granite gneiss	77° 3’ 34.596“E 9° 45’ 6.48“N	780	2500	21.6	
WCP	Teak	Hornblende-biotic gneiss	76° 1’ 20.316’‘ E 11° 54’ 4.24’‘ N	752	2659	22.6	Ultisol
WCP	Forest	Hornblende-biotic gneiss	76° 1’ 17.22’‘ E 11° 54’ 0.36’‘ N	754	2659	22.6	
NCL	Teak	Charnockite	76° 21’ 7.16’‘ E 10° 39’ 11.41’‘ N	259	2800	27.5	Ultisol
NCL	Forest	Charnockite	76° 20’ 58.38’‘ E 10° 38’ 44.268’‘ N	250	2790	27.5	
NHH	Teak	Charnockite	76° 21’ 17.424’‘ E 11° 16’ 58.26’‘ N	907	2200	27.6	Inceptisol
NHH	Forest	Charnockite	76° 21’ 5.076’‘ E 11° 16’ 57.144’‘ N	901	2200	27.6	

*Based on USDA soil classification

Southern Central Foot hills (SCF), Southern High Hills (SHH), Wayanad Central Plateau (WCP), North Central Laterites (NCL), Northern High Hills (NHH), Teak Plantation (Teak) Natural Forest (Forest)

The SHH, primarily in Idukki District, covers 672,675 ha (17.31%) and features steep slopes, lower temperatures, and a mean annual precipitation of 3602 mm. The SCF, spanning several districts, including Kollam and Pathanamthitta, encompasses 315,893 ha (8.13%) of undulating lands with low hills. Soil samples were collected from two representative sites within this region and combined to form the composite SCF sample set. The North Central Laterites region covers 171,469 ha (4.41%) in the State [17].The lowlands have strong acid, non-gravelly clay soils with impeded drainage, and the upland shave strongly acid, gravelly, lateritic, low-activity clay soils, often underlined by plinthite. Kerala’s Northern High Hills agro-ecological unit encompasses parts of Malappuram and Kannur districts, covering 5,28,434 hectares (13.60% of the state). The soils in this region are predominantly deep, well-drained lateritic soils with clay to loam texture, slightly acidic to neutral in reaction, and well-supplied with bases. The Wayanad Central Plateau agroecological unit is a distinct region in Kerala characterized by its unique soil and climatic conditions. The climate of this region is tropical humid monsoon type, with a mean annual temperature of 22.6°C and an average rainfall of 2659 mm. The uplands of the Wayanad Central Plateau contain deep, acidic clay soils that are rich in organic matter.

In each zone, we selected adjacent natural forests (moist deciduous) and teak plantations (*Tectona grandis*) for our study. The natural forests featured diverse species, including *Euvodia lunuankenda, Artocarpus heterophyllus, Calophyllum elatum, Bischofia javanica, Mesua ferrea, Mangifera indica, and Myristica dactyloides*,*Tabernaemontana heyneana, Dillenia pentagyna, Strychnos nux-vomica, and Xylia xylocarpa*. The teak plantations were selected in the age group 40–50 years as silvicultural operations in teak are over by 30 years, and thereafter, the soil is left undisturbed till final felling. Further, this provided a 90–100 years of continuous teak growth after conversion of natural forests. These soils are typical of humid tropical regions and are characterized by clay-rich horizons with low nutrient availability. The climate is categorized as Am (Tropical Monsoon) under the Köppen Climate Classification.

### 2.2. Soil sample collection

We collected soil samples from natural forest sites and continuous teak-planted areas adjacent to these forests. We employed a multistage random sampling method, using geological parent material as the main strata and forest type and agro-ecological units as substrata. This approach allowed us to identify significant teak-growing areas alongside natural forests in the Western Ghats of Kerala. We determined the geological classification based on the mineral and chemical composition, permeability, texture, and particle size of the parent material (rocks).

Soil sampling was conducted using 1-meter-deep excavation pits in teak plantation and adjacent natural forest ecosystems. A minimum of three pits per forest system were established, with final counts determined by site-specific conditions. Sampling followed a toposequence approach, with three designated points per study area. At each point, pits were dug to 1-meter depth to collect specimens from four genetic horizons: O (organic), A (topsoil), B (subsoil), and C (substratum). Samples were collected from a minimum three points located 50 m apart in each plantation and forest, mixed to get composite samples. This design yielded 144 total samples, representing 12 forest types × 3 patches × 4 horizons. Genetic horizon samples were sieved through 2 mm mesh prior to laboratory processing.

### 2.3. Analytical methods

Soil samples were prepared by removing root fragments, air-drying, grinding, and sieving through a 2 mm sieve for subsequent physical and chemical analyses. Bulk density was estimated using the core method (volumetric cylinder method) based on the mass–volume relationship, with the following equation:


Bulk density (Mg/m³) = Weight of dried soil (Mg) / Soil volume (m³)

Soil color was identified using the Munsell color chart. Soil texture was determined by the hydrometer method [18]. Soil pH was measured in a soil:water suspension ratio of 1:2.5 using potentiometry [19]. Cation exchange capacity (CEC) was assessed using the neutral normal NH_4_OAc (pH 7.0) saturation method, and the percentage of base saturation (BS) was calculated as the ratio of basic ions to CEC [20]. Total soil organic carbon (SOC) content was estimated in the soil samples using a CHNS analyzer (Eurovector EA3000). The soil organic carbon (SOC) stock were calculated using the equation [21]


$$\text{Cm}\;=\sum_{i=\text{1}}^{n}\text{B}\text{i}*\text{TC}\text{i}*\text{di}*(\text{1}-\text{Si})$$

where Cm = Carbon stocks (Mg m^-2^).,

TCi = Total carbon of i^th^ layer (g C/ g), Bi = Bulk density of i^th^ layer (Mg m^-3^), di = Depth of i^th^ layer (m), Si = volume proportion of fragments > 2 mm.

### 2.4. Statistical analysis

The analysis examines the relationship between forest and teak plantation land-use types with respect to soil organic carbon (SOC) and soil properties. Data normality was assessed using the Shapiro-Wilk test, and homogeneity of variances was tested with Levene’s test. A Two-way ANOVA was performed to evaluate differences in soil properties and SOC between natural forests and teak plantations across various soil depths. Mean comparisons utilized the least significant difference (LSD) test at a 5% significance level (p < 0.05). Pearson’s correlation analysis was also conducted to explore relationships between SOC concentration and selected soil properties.

All statistical analyses in the study were performed using R (version 4.1.2) and SPSS (version 19.0) software packages. To ensure the reliability of the results, each soil sample was analyzed in triplicate and the graphical representations of the data were created using Origin 2018 and CorelDRAW X4 software.

## 3. Results and discussions

### 3.1. Physicochemical properties of soil in forest systems and teak plantation systems

An assessment of soil properties in natural forests and teak plantations across the different agro-ecological units of the Kerala region in the Southern Western Ghats shows clear differences in their physical and chemical characteristics. These contrasts help explain how shifts in land use influence the carbon-sequestration capacity of tropical forest soils. Table 2 summarizes the morphological, textural, and chemical features of the soil horizons examined in natural forests and teak plantations within the study area.

**Table 2:** Comparative assessment of textural and chemical properties soil properties of natural forest and teak planted systems in different agro-ecological units.

Agro-ecological Unit		Colour	Sand (%)	Silt (%)	Clay (%)	Soil texture	Bulk density (Mg/m^3^)	Moisture%	pH	Electrical conductivity (dS/m)	CEC (cmol(+)kg-1 soil)	Base saturation (%)	Al^3+^
**Natural forest**													
SCF		5YR 3/2	80	2	18	*sl*	1.55	7.21	5	0.06	25.38	32.45	10.0
SHH		5YR 3/2	70	8	22	*scl*	1.22	13.64	4.9	0.05	23.00	32.00	12.02
WCP		5YR 2.5/2	75	4	20	*scl*	1.35	8.2	5.3	0.02	32.45	31.3	10.21
NCL		5YR 3/2	68	10	22	*scl*	1.21	11.2	5.79	0.04	24.00	34.05	7.30
NHH		5YR 2.5/2	78	6	16	*sl*	1.26	12.1	6.01	0.06	22.23	30.92	6.25
**Teak Plantation**													
SCL	10YR 4/4		74	2	24	*scl*	1.43	6.05	4.8	0.05	48.18	35.77	12.0
SHH	5YR 5/4		68	8	24	*scl*	1.11	15.19	4.5	0.03	23.40	23.59	11.0
WCP	5YR 4/4		75	5	20	*scl*	1.24	12.1	4.9	0.04	39.12	32.1	11.21
NCL	5YR 5/4		76	8	16	*sl*	1.59	14.1	5.73	0.02	22.2	31.99	13.0
NHH	5YR 4/4		74	4	22	*scl*	1.21	12.2	5.39	0.06	16.04	28.24	7.25

sl-sandy loam texture, scl- sandy clay loam texture.

In warm, humid tropical climates where wet and dry seasons alternate, laterite soils commonly develop. Strong weathering and continuous leaching remove mobile base cations such as Ca, Mg, K, and Na, while less soluble compounds of iron, aluminium, and silica accumulate. As sesquioxides become concentrated and basic salts are depleted, the soils become strongly acidic, often registering pH values below 7. In Kerala, soils are predominantly acidic laterites, typically ranging from pH 4.09 to 6.22 [22]. In this study as well, soil acidity was evident at all locations, with pH values between 5.79 in natural forest soils and 4.90 in teak plantations. This pattern is consistent with observations from other humid tropical environments, where intense leaching drives soil acidification [23].

Within the North Central Laterites (NCL), both natural forests and teak plantations showed sticky upper horizons. Clay content remained fairly uniform throughout the soil profile under natural forests, whereas soils under teak plantations exhibited clay illuviation in deeper layers. These differences in clay movement suggest that teak plantation management influences soil-forming processes. Additionally, the presence of argillic horizons in the B layers of natural forest soils highlights the distinct pedogenic development occurring in these ecosystems. The stronger acidity observed in teak plantation soils is largely due to the greater quantity of litter they produce and its rapid decomposition, which releases weak organic acids into the soil environment [24]. Clear differences also appeared in cation exchange capacity (CEC) between land-use systems. Natural forest soils showed wider CEC values (15.60–24 cmol (+) kg ⁻ ¹) across horizons, while teak plantations had slightly lower and more restricted values (17.40–22.20 cmol (+) kg ⁻ ¹). Within the NCL region, soils under teak exhibited higher concentrations of exchangeable Al³⁺ than those under natural forests. The elevated base saturation noted in the C horizon of teak plantations is likely a consequence of reduced CEC rather than an actual increase in basic cations.

In the Southern Central Foothills (SCF), prolonged teak cultivation promoted the formation of argillic horizons, and base saturation declined to below 35% throughout the profile (Table 3). Repeated teak rotations also intensified soil acidity; surface layers that were moderately to strongly acidic in natural forests (pH 5.28–5.87) shifted to strongly acidic conditions (pH 4.59–4.98). Soils in the Southern High Hills further demonstrated pronounced textural contrasts between the two systems. Natural forest profiles retained a consistent sandy clay loam texture, whereas teak plantation profiles displayed noticeable stratification—fine sandy clay loam near the surface grading into sandy loam at depth. Plantations also exhibited reduced surface bulk density, which may reflect changes in soil aggregation and organic matter inputs [25]. Teak cultivation in this region caused a marked decline in surface pH to 4.5–5.0, indicating a more acidic environment than that of adjacent forest soils.

**Table 3:** Selected Horizon wise textural and chemical properties of natural forest and teak planted systems.

Agro-ecological Unit	Horizon	Colour	Sand (%)	Silt (%)	Clay (%)	Soil texture	pH	CEC (cmol(+)kg-1 soil)	Base saturation (%)	Al^3+^
SCF-Forest	A	5YR 3/2	80	2	18	sl	5.00	25.38	32.45	10.0
	B	5YR 3/3	72	4	24	scl	5.36	17.29	33.30	11.0
	C	5YR 4/6	66	4	30	scl	5.28	23.51	33.88	11.0
SCF-Teak	O	10YR 4/4	74	2	24	scl	4.80	48.18	34.77	12.00
	A	5YR 5/6	61	5	34	scl	4.83	47.26	34.10	14.10
	B	7.5YR 5/6	62	4	34	scl	4.59	43.14	34.14	13.00
	C	7.5YR 5/6	69	9	23	scl	5.30	46.99	34.94	11.00
SHH-Forest	O	5YR 3/2	70	8	22	scl	4.90	23.00	32.00	12.02
	A	5YR 3/3	69	7	24	scl	5.84	15.20	33.80	20.04
	B	2.5YR 3/4	69	8	23	scl	5.82	14.04	21.64	19.80
	C	2.5YR 4/6	64	10	26	scl	5.71	7.68	18.78	21.02
SHH-Teak	O	5YR 5/4	68	8	24	scl	4.50	23.40	23.59	11.00
	A	5YR 4/4	70	8	22	scl	4.52	23.02	21.14	22.10
	B	5YR 5/8	72	6	22	scl	5.46	7.58	20.28	22.00
	C	7.5YR 5/6	74	8	18	sl	5.50	6.13	10.37	10.76
WCP-Forest	O	5YR 2.5/2	75	4	20	scl	5.30	32.45	31.3	10.21
	A	5YR 2.5/2	72	6	22	scl	5.31	25.62	30.21	15.62
	B	5YR 2.5/2	73	8	19	sl	5.28	24.12	25.42	18.12
	C	5YR 3/2	69	7	24	scl	5.45	15.12	20.12	12.21
WCP-Teak	O	5YR 4/4	75	5	20	scl	4.90	39.12	32.1	11.21
	A	5YR 4/4	70	5	25	scl	4.84	30.12	30.21	10.21
	B	5YR 4/4	71	3	26	scl	4.95	20.12	25.12	11.25
	C	5YR 4/4	69	4	27	scl	5.32	15.21	16.12	12.12
NCL-Forest	O	5YR 3/2	68	10	22	scl	5.79	24.00	34.05	7.30
	A	5YR 3/3	66	8	26	scl	5.20	26.20	34.94	11.21
	B	2.5YR 3/4	66	8	26	scl	5.20	15.60	31.78	12.01
	C	2.5YR 4/6	62	12	26	scl	5.02	20.00	30.72	13.20
NCL-Teak	O	5YR 5/4	76	8	16	sl	5.73	22.20	31.99	13.0
	A	5YR 4/4	72	6	22	scl	5.65	17.4	31.92	12.2
	B	5YR 5/8	70	6	24	scl	5.40	22.6	33.11	12.2
	C	7.5YR 5/6	67	4	29	sl	4.90	18.12	34.51	13.1
NHH-Forest	O	5YR 2.5/2	78	6	16	sl	6.01	22.23	30.92	4.10
	A	5YR 3/2	70	8	22	scl	5.94	20.81	28.71	7.04
	B	5YR 3/2	72	10	18	sl	5.85	13.22	22.83	3.02
	C	5YR 3/4	60	5	35	scl	5.78	7.64	21.72	3.01
NHH-Teak	O	5YR 4/4	74	4	22	scl	5.39	16.04	28.24	4.22
	A	5YR 4/4	66	6	28	scl	5.08	12.42	28.35	7.30
	B	5YR 4/4	64	6	30	scl	4.88	10.23	11.32	5.02
	C	5YR 4/4	62	6	32	scl	5.00	16.04	28.24	3.02

sl-sandy loam texture, scl- sandy clay loam texture.

Across the Northern High Hills (NHH), both land-use types remained acidic. Natural forest soils featured sticky B horizons and well-developed argillic horizons, with textures ranging from sandy clay loam to sandy loam. Overall, the combined assessment of soil morphological, textural, and chemical traits across the agro-ecological units demonstrates consistent shifts following the conversion of natural forests to teak plantations. These changes—enhanced acidity, modified clay redistribution, and altered organic-matter characteristics—underscore the substantial impact of land-use transition on soil development [26]. The results emphasize the need for sustainable management strategies in tropical forest landscapes to safeguard soil health and to strengthen their capacity for long-term carbon sequestration [27].

### 3.2. Characteristics of Soil organic carbon distribution in forests and teak plantation systems

We analyzed SOC content within the 0–100 cm soil profile in both natural forest and teak plantation systems, with the results presented in Table 4. Across all sites, the average SOC was 16.61 g/kg in natural forests and 11.82 g/kg in plantations. Compared to adjacent forests, SOC in teak plantations was lower by 52% at NCL, 97% at SHH, 37% at WCP, and 22% at NHH. Interestingly, at SCF, the teak plantation showed a 46% higher SOC than the corresponding natural forest, representing an exception to the general trend. Overall, SOC concentrations were consistently higher in natural forests at NCL, SHH, WCP, and NHH compared to plantations.

**Table 4:** Soil Carbon (SOC) stocks and SOC (t C ha ⁻ ¹) by site and forest type.

Study Region	Mean SOC content ± ^*^SE (g/kg)(m)	CV (%)	Mean Soil Carbon stock ± ^*^SE (t C ha ⁻ ¹)	CV (%)
SCF-Teak	17.73 ± 0.11B^a^	2.4	438.95 ± 0.50B^a^	2.5
SCF- Forest	12.11 ± 0.11B^b^	2.6	348.52 ± 0.80B^b^	1.7
SHH- Teak	11.54 ± 0.04C^a^	3.6	185.11 ± 0.22C^a^	1.4
SHH- Forest	26.09 ± 0.18C^b^	2.8	395.96 ± 1.00C^b^	1.7
WCP-Teak	16.49 ± 0.07D^a^	3.6	195.49 ± 0.50D^a^	3.1
WCP-Foresr	20.84 ± 0.09D^b^	4.9	413.30 ± 0.80D^b^	2.3
NCL-Teak	6.93 ± 0.05A^a^	3.9	148.38 ± 0.39A^a^	3.1
NCL-Forest	14.49 ± 0.27A^b^	3.5	354.26 ± 1.00A^b^	3.4
NHH-Teak	7.41 ± 0.07A^a^	6.5	163.95 ± 0.50A^a^	3.6
NHH-Forest	9.53 ± 0.08A^b^	5.3	318.88 ± 0.80A^b^	3

Values are presented as mean ± standard error (SE); *n* = 144 per site and ecosystem type.

*SE (g/kg)(m): Standard error of mean SOC concentration over soil depth (m).

*CV: Coefficient of variation, expressed as a percentage.

*Lowercase superscript letters denote significant differences between ecosystem types (teak vs. natural forest) within the same site (*P* < 0.05, LSD test).

* Uppercase superscript letters denote significant differences among sites within the same ecosystem type (*P* < 0.05, LSD test). Letter assignments are based on actual LSD post-hoc test results

According to earlier studies conducted in tropical climates, natural forests have a higher soil organic carbon (SOC) storage capacity than plantations. For instance, in NCL, the SOC concentration in natural forests (14.49 g/kg) was double that in plantations (6.93 g/kg). Studies addressed how different land uses differ in the Western Ghats, demonstrating that natural forests have a higher SOC sequestration capacity than monoculture crops such as rubber and teak. Similarly, tropical forests in southern India have higher SOC storage due to their diversified vegetation and robust ecosystem processes [28] and these findings are supported by [29,30]. Recent studies also show that, in terms of both the quality and quantity of carbon capture, forests are superior and cannot be replaced by plantations as moderators of terrestrial carbon sequestration, especially during drought periods [31]. These studies emphasize the observation that natural forests are more successful at storing carbon in soil than teak plantations, highlighting the importance of evaluating land use patterns that enhance SOC storage while also supporting sustainable carbon management strategies. Natural forests have a complex plant diversity and understory, which contributes to increased organic matter input and microbial activity in the soil, whereas teak plantations exhibit lower SOC storage due to less diverse vegetation caused by monoculture environments [32]. This observation aligns with [33] findings on the importance of soil organic matter in tropical forest carbon cycles. Our results support this, showing significantly higher SOC concentrations at SHH (26.09 g/kg in forests versus 11.54 g/kg in plantations) and WCP (20.84 g/kg versus 16.49 g/kg).

**Fig 2:**
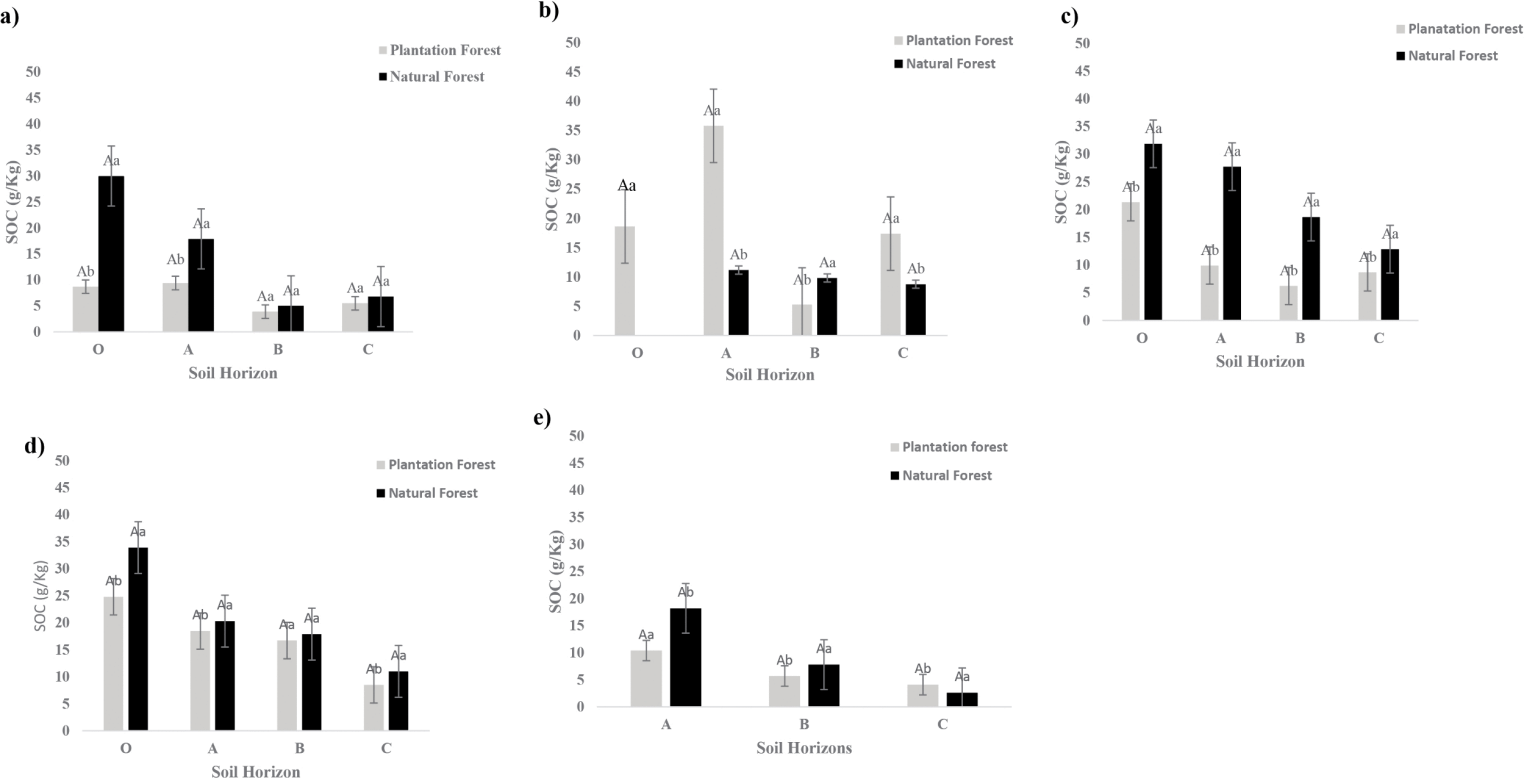
Variation in SOC distribution across soil profiles of plantation and natural forests at (a) NCL, (b) SCF, (c) SHH, (d) WCP, and (e) NHH sites. Significant differences between forest types are indicated by different uppercase letters (P < 0.05), while differences across soil layers are marked by distinct lowercase letters (P < 0.05).

Fig 2 (a-e) illustrates the proportion of soil organic carbon (SOC) stored in each soil horizon across different land-use types, showing the percentage contribution of individual horizons to the total SOC stock in both natural forests and teak plantations at each site. This comparison highlights the relative roles of surface and subsoil layers in carbon storage and supports the patterns observed in our SOC measurements. Surface horizons (O and A) generally contain the highest SOC concentrations, which decline with depth, although the rate of decrease varies depending on land use and site characteristics. In natural forests, 65–85% of total SOC is concentrated in the O and A horizons, whereas teak plantations store a smaller fraction at the surface, ranging from 50–70%. At SCF, surface SOC in teak plantations is unusually high, likely reflecting prolonged management and longer rotation periods. The C horizon consistently contributes a minor portion of total SOC, usually less than 5–10%. These results emphasize the strong influence of land-use type and management practices on the vertical distribution of SOC. In Kerala, studies such as [28] emphasize that monoculture teak plantations contribute less to carbon storage than natural forests, primarily due to vegetation diversity and soil management differences [33]. Study also highlighted that tropical dry forests have higher carbon stocks compared to teak plantations. However, some studies have reported that teak plantations may exhibit comparable or higher SOC storage depending on site conditions and management practices [34]. For example, mature teak plantations have been shown to reach over 230 t ha ⁻ ¹ carbon stocks [35].

The teak plantations, especially older ones, can accumulate substantial SOC amounts, at times exceeding degraded forests [36,27]. Plantation age and management are critical, with older plantations (over 50 years) accumulating more SOC [37]. In northeastern India, well-managed teak plantations achieved SOC levels similar to natural forests, particularly where natural forests are disturbed or in early succession [38]. The higher SOC concentration in SCF plantations (17.73 g/kg vs 12.11 g/kg) correlates with longer rotation cycles and management such as thinning and litter decomposition. These observations are supported by [39] who noted well-managed teak plantations accumulate more litter and SOC than degraded forests. Additional research [34] shows teak plantations can mitigate climate change by sequestering soil carbon and gradually improving SOC on degraded lands. Moreover, plantations planted on degraded or deforested lands enhanced soil quality and SOC [40]. Thus, while natural forests generally store more SOC, well-managed plantations can accumulate substantial amounts.

The relatively high SOC concentrations in teak plantations indicate that effective management and regulated rotation in Kerala significantly contribute to carbon accumulation. [41] reported that productivity and carbon inputs of man-made forests often surpass those of natural forests. Beyond productivity, plantation forests can also contribute to biodiversity conservation, providing habitat for avian species in fragmented landscapes and offering refuge for understory flora typically found in secondary forests [29].

Tree species composition plays a key role in determining carbon sequestration potential. *Tectona grandis* (teak), with its rapid growth and high biomass accumulation, can sequester carbon efficiently over time, contributing to soil carbon storage [42]. In our study, SOC patterns in plantations followed the order NCL < NHH < SHH < WCP < SCF, differing from natural forests that followed NHH < SCF < NCL < WCP < SHH concentrations in natural forests ranged from 9.53 to 26.09 g/kg, compared to 6.93–17.73 g/kg in teak plantations, confirming that natural forests generally maintain higher soil carbon levels.

Regional climate also influences SOC stability. In Humid tropical zones, high precipitation combined with slopping terrain promotes leaching of dissolved organic carbon. Sandy soils with low water-holding capacity and intense rainfall further exacerbate SOC loss. For instance, the Agasthyamalai Hills (NCL) receive over 2800 mm of rainfall annually, contributing to substantial leaching and lower SOC concentrations in both plantation and forest soils. As summarized in Table 4 and Fig 2, natural forests exhibited higher carbon stocks than teak plantations at four of the five sites (NCL, SHH, WCP, NHH), consistent with global meta-analyses indicating that converting forests to plantations reduces SOC by roughly 13% [43,44]. This decline is likely driven by changes in litter quality, reduced root biomass inputs, soil disturbance during planting, and decreased microbial diversity in monocultures. Notably, at SCF, teak plantations contained 21% higher SOC than natural forests, deviating from the general trend. Such exceptions typically occur when plantations are established on degraded land or managed to enhance soil inputs while minimizing disturbances [44]. SOC variability, measured by the coefficient of variation (CV), was relatively low within ecosystem types (1.4–3.6%), suggesting consistent carbon stock distribution at the plot scale. This supports the conclusion that land-use type exerts a stronger influence on SOC than microsite variability, in agreement with [44]. Beyond carbon storage, natural forests also provide biodiversity and ecosystem resilience benefits. These results reinforce the importance of protecting and restoring native forests as a priority strategy for climate mitigation. However, in regions where natural forests have been lost or severely degraded, well-managed plantations can act as transitional systems to increase carbon storage, provided they incorporate diverse species, maintain soil cover, and mimic natural ecological processes.

### 3.3. Connecting SOC concentration to soil properties: Soil pH as a factor in SOC variation

Soil pH is a subtle yet influential factor in shaping soil organic carbon (SOC) dynamics. Although its effects may not be immediately apparent, even slight changes in pH can alter the entire carbon cycle in the soil. In our study region in the Southern Western Ghats, soil pH ranged from 4.5 to 6.01, indicating strong to slight acidic soil. Interestingly, we observed a discernible trend in O horizon - pH variation across systems, following the order: NHH-F > NCL-F > NCL-T > NHH-T > WCP-T > SCF-F > WCP-F > SCF-T > WCP-T > SHH-T, with significant differences (P < 0.05). When comparing natural forest and teak plantation systems, the pH values in natural forests (NCL-F: 5.79, SCF-F: 5.0, SHH-F: 4.9, WCP-F: 5.3, NHH-F: 6.01) were generally higher than those in plantation soils (NCL-T: 5.73, SCF-T: 4.8, SHH-T: 4.5, WCP-T: 4.9, NHH-T: 5.39). In teak plantations, the breakdown of organic acids from teak litter can result in the production of acidic compounds (Humic acids), which temporarily lower soil pH. Over time, monoculture plantations may experience greater acidity due to the higher rate of litter input and decomposition [34].

**Fig 3:**
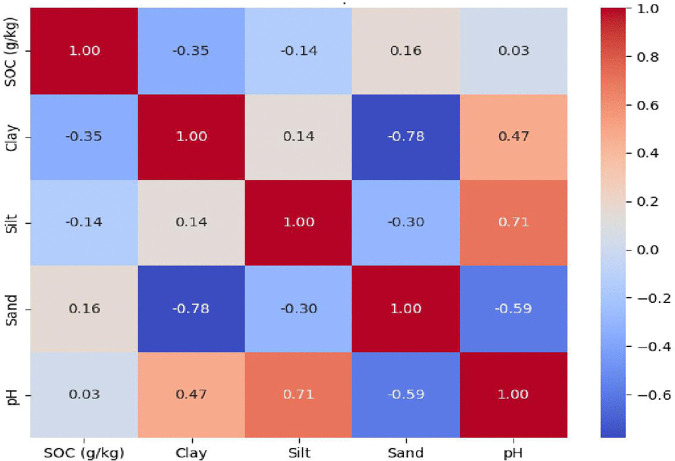
Pearson correlation heatmap showing relationships between soil properties in Teak plantations.

Additionally in plantations, fertilizers, especially those with ammonium or other acidic compounds, are used to improve growth, which can acidify soils. Lime is sometimes added to counteract acidity, but if not applied sufficiently, soil pH remains relatively low. The fertilizers commonly used in teak plantations in Kerala, such as nitrogen (N), phosphorus (P), potassium (K), balanced NPK formulations (e.g., 15:15:15), lime, and magnesium, can affect soil pH in different ways. Ammonium-based N fertilizer and P fertilizer acidify the soil and lower its pH. Additionally, balanced NPK (15:15:15) includes potassium and phosphate salts together with ammonium or urea as a source of nitrogen [45,46]. Over time, it usually causes soil to become more acidic due to the ammonium it contains [45]. Interestingly, in our study, teak planted soils shows as positive correlation (r = 0.03) with SOC accumulation Fig 3, which contradicts earlier studies suggesting that alkaline soils are more prone to SOC losses due to reduced complexation of organic matter on mineral surfaces through ligand exchange mechanisms. Lu et al. (2023) reported that in acidic soils, the formation of humic acids is enhanced, which reduces microbial biomass and slows the decomposition of SOC by microbes [47].

**Fig 4:**
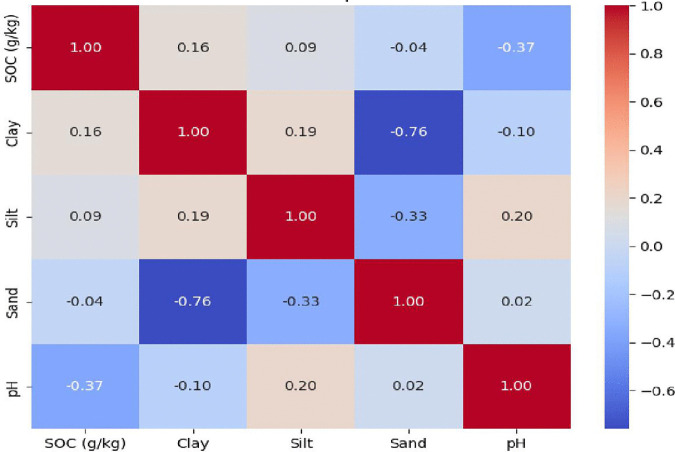
Pearson correlation heatmap showing relationships between soil properties in natural forests.

Typical forest soils in humid tropical climates are generally acidic due to the leaching of base cations and the release of organic acids from the decomposition of abundant organic matter, as well as uptake of cations by diverse vegetation. In forest ecosystems, trees often take up base cations in excess of accompanying anions to meet physiological demands for growth. To maintain charge balance, roots release protons (H⁺) into the rhizosphere, causing soil acidification. In addition, the return of relatively more acidic litter and throughfall further reinforces this process [48]. Over time-particularly in highly weathered tropical soils with limited base inputs-this net proton release leads to progressive soil acidification [49]. Fig 4 illustrates the Pearson correlation analysis between pH (acidic, neutral, or alkaline) and SOC accumulation. It is well established that pH can influence soil structure, water retention, aeration, and microbial activity in the rhizosphere—factors that collectively impact SOC dynamics through organic matter decomposition and formation of stable soil organic carbon [50]. Consistent with these findings, our results also showed a significant negative correlation between pH and SOC accumulation in forest ecosystem (−0.37).

### 3.4. Soil texture as a factor in SOC variations

The Wilcoxon Rank Sum Test indicated no statistically significant differences in soil particle size distribution between natural forest and teak plantation systems, with sand (U = 15.0, p = 0.30), silt (U = 14.5, p = 0.47), and clay content (U = 8.0, p = 0.57) all yielding p values greater than 0.05.Here U denotes the test statistic based on rank sums, and p represents the corresponding probability value used to assess statistical significance (p < 0.05).Nevertheless, descriptive analyses revealed some site-specific differences. Sand content was generally high across all locations, ranging from 60–80% in natural forests and 61–76% in teak plantations. Silt content remained low, varying from 2–12%, with the highest silt proportion (12%) in the C horizon of the NCL natural forest. Clay content across all horizons ranged from 16–35%, with slightly greater variability in plantation soils. The highest surface clay content (24%) occurred in teak plantations at SHH and SCL, whereas the highest overall clay content was observed in the C horizon of the NHH natural forest (35%). Despite these minor differences, soil texture classes remained predominantly sandy loam (sl) or sandy clay loam (scl) in both systems, indicating that conversion from natural forests to plantations did not substantially alter fundamental soil texture. Heatmaps (Figs 3 and 4) illustrate correlations among SOC, clay, silt, sand, and pH. The color scale ranges from −1.0 to 1.0, with dark blue indicating a strong negative correlation and dark red indicating a strong positive correlation. Correlation analysis of SOC with soil textural components revealed contrasting patterns between natural forests and teak plantations. In plantations, SOC showed a negative correlation with silt (−0.14) and clay (−0.35), but a positive correlation with sand (0.16), suggesting that SOC accumulation does not always align with clay content as traditionally expected. In natural forests, SOC exhibited a positive relationship with clay (0.16) and silt (0.09), a mild inverse relationship with sand (−0.04) The highest surface SOC values (30–35 g/kg) were associated with relatively high clay contents (20–25%). Positive SOC-sand correlations in plantations may appear counterintuitive, as clay particles generally stabilize organic matter through aggregation and physicochemical protection [51]. However, these patterns are influenced by additional factors, including soil aggregation, microbial diversity, vegetation inputs, and management practices. In acidic plantation soils, elevated exchangeable Al³ ⁺ competes with Ca²⁺ for mineral binding sites, leading to the formation of organo-mineral complexes that are less effective in promoting aggregation [52]. In addition, Al³ ⁺ toxicity suppresses root growth as well as microbial biomass and enzyme activity, thereby reducing belowground carbon inputs and microbial processing of fresh organic matter into stable mineral-associated SOC; consequently, SOC accumulation in horizons is often constrained, even in clay-rich soils [53]. In Kerala teak plantation the negative correlation of SOC and clay, likely due to Al-dominated subsoil chemistry. Aluminum (Al³⁺) concentrations increased with depth – being higher in the A, B, and C soil horizons and lower at the surface (O horizon) across all sites and were generally higher in teak plantations compared to natural forests, mirroring observed pH declines. In natural forests there was a strong negative correlation between clay and sand (−0.76), a mild inverse relationship with pH (−0.10), and a weak positive correlation with silt (0.19). In teak plantations, Clay had a moderate positive correlation with pH (0.47). These variations significantly influence carbon storage capacities and overall ecosystem functioning, underscoring the importance of site-specific soil management strategies [54]. These variations emphasize the distinct soil dynamics and carbon storage capacities between natural and plantation ecosystems.

**Fig 5:**
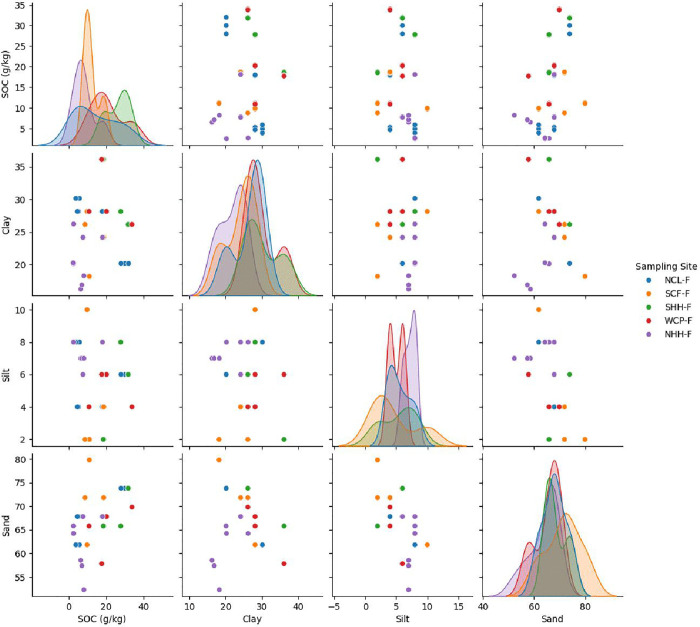
Pairwise plots showing relationships among soil organic carbon (SOC), clay, silt, and sand across five Kerala forest sites.

**Fig 6:**
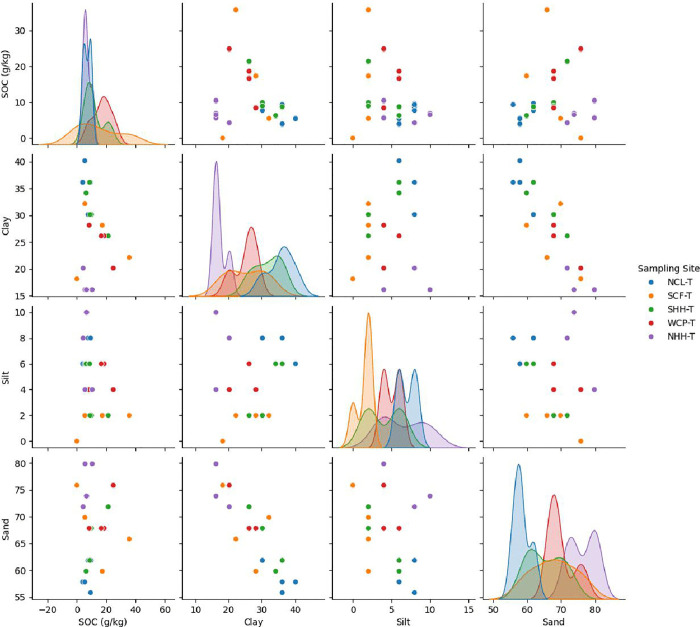
Pairwise plots showing relationships among soil organic carbon (SOC), clay, silt, and sand across five Kerala Teak plantation sites.

Pair plots (Fig 5) (Fig 6) further reveal site-specific patterns. The diagonal plots illustrate the distribution of each variable by site and the off-diagonal scatterplots display their pairwise interactions. In site specific forest soils SOC generally increases with higher clay and lower sand, supporting clay’s role in organic matter stabilization. These findings align with earlier reports on forest soil carbon dynamics and emphasize the importance of both soil texture and local conditions in influencing SOC pools. In plantations, SOC-clay relationships were more variable, reflecting influences from plantation age, management practices, and human disturbances. Teak monocultures, for instance, can produce lignin-rich litter that decomposes slowly but may not integrate efficiently with clay minerals due to limited microbial diversity and suboptimal pH. This can result in lower SOC retention despite high clay content. Overall, human activity in Southern Western Ghats plantations tends to disrupt natural soil processes, creating sand-dominated surface layers and clay/silt-enriched subsoils, in contrast to the more stable and organically regulated texture profiles of natural forests [55,56].

## 4. Conclusion

This study demonstrates that soil organic carbon (SOC) dynamics in the Southern Western Ghats are strongly influenced by land-use type, soil texture, chemistry, and management history. Natural forests consistently maintained higher SOC concentrations compared to teak plantations (except at the Southern Central Foothills, where long rotation cycles (>40 years) and minimal disturbance allowed plantations to accumulate higher SOC. The findings underscore the importance of improving plantation management to enhance SOC retention. Strategies include extending rotation lengths, minimizing soil disturbance, implementing erosion control measures, integrating agroforestry systems, and applying clay-organic amendments. These interventions can help bridge the SOC gap between plantations and natural forests, improve soil microbial activity, and strengthen ecosystem services. Policymakers and land managers should prioritize sustainable management and restoration strategies to prevent the creation of “carbon false sinks” and maintain resilient soil carbon stocks. With appropriate practices, teak plantations can gradually transition toward functioning as effective carbon reservoirs, contributing to local soil health and broader climate change mitigation.
